# The role of mitochondrial DNA copy number in autoimmune disease: a bidirectional two sample mendelian randomization study

**DOI:** 10.3389/fimmu.2024.1409969

**Published:** 2024-10-09

**Authors:** Zhekang Liu, Qingan Fu, Yijia Shao, Xinwang Duan

**Affiliations:** ^1^ Rheumatology and Immunology Department, The Second Affiliated Hospital of Nanchang University, Nanchang, Jiangxi, China; ^2^ Cardiovascular Medicine Department, The Second Affiliated Hospital of Nanchang University, Nanchang, Jiangxi, China

**Keywords:** mitochondrial DNA copy number, autoimmune disease, Mendelian randomization, causal association, GWAS

## Abstract

**Background:**

Mitochondrial DNA (mtDNA) plays an important role in autoimmune diseases (AD), yet the relationship between mitochondria and autoimmune disease is controversial. This study employed bidirectional Mendelian randomization (MR) to explore the causal relationship between mtDNA copy number and 13 ADs (including ankylosing spondylitis [AS], Crohn’s disease [CD], juvenile rheumatoid arthritis [JRA], polymyalgia rheumatica [PMR], psoriasis [PSO], rheumatoid arthritis [RA], Sjogren’s syndrome [SS], systemic lupus erythematosus [SLE], thyrotoxicosis, type 1 diabetes mellitus [T1DM], ulcerative colitis [UC], and vitiligo)

**Methods:**

A two-sample MR analysis was performed to assess the causal relationship between mtDNA copy number and AD. Genome-wide association study (GWAS) for mtDNA copy number were obtained from the UK Biobank (UKBB), while those associated with AD were sourced from the FinnGen Biobank. Inverse variance weighting (IVW) was the primary analysis method, complemented by three sensitivity analyses (MR-Egger, weighted median, weighted mode) to validate the results.

**Results:**

IVW MR analysis identified significant associations between mtDNA copy number and CD (OR=2.51, 95% CI 1.56-4.22, P<0.001), JRA (OR=1.87, 95% CI 1.17-7.65, P=0.022), RA (OR=1.71, 95%CI 1.18-2.47, P=0.004), thyrotoxicosis (OR=0.51, 95% CI0.27-0.96, P=0.038), and T1DM (OR=0.51, 95% CI 0.27-0.96, P=0.038). Sensitivity analyses indicated no horizontal pleiotropy.

**Conclusions:**

Our study revealed a potential causal relationship between mtDNA copy number and ADs, indicating that these markers may be relevant in exploring new therapeutic approaches.

## 1 Introduction

Autoimmune diseases (ADs) are characterized by the immune system mistakenly attacking the body’s own tissues, leading to chronic inflammation and tissue damage (1). The burden of ADs on patients is substantial, encompassing a wide array of chronic conditions that significantly impair quality of life, lead to long-term health care needs, and pose considerable challenges to health care systems worldwide (2).

In the context of ADs, the interplay between mitochondrial dynamics and immune responses is an area of intense investigation. Mitochondria, the powerhouses of the cell, are central to energy production and are intimately involved in a myriad of cellular processes, including apoptosis, signaling, and reactive oxygen species production (3). Their role extends beyond mere energy suppliers to being pivotal in maintaining cellular homeostasis and function. The number of mitochondria within a cell, quantified as the mitochondrial DNA (mtDNA) copy number, reflects the cell’s metabolic demand and its ability to respond to physiological stress (4). This metric has emerged as a significant biomarker for various diseases, highlighting the intricate relationship between mitochondrial function and pathological states (5). A number of previous cross-sectional studies, case-control studies reported an increased risk of developing some ADs has been reported to be associated with a reduction in mitochondrial copy number (6–9). Several observational studies have reported that mtDNA is associated with AS (10), CD, UC (11), PSO (12). To date, it is unclear whether there is a causal relationship between mtDNA copy number and these autoimmune diseases. Furthermore, it has yet to be determined whether a bidirectional relationship exists, whereby autoimmune disease might influence mtDNA copy number.

MR is an epidemiological approach based on genome-wide association study (GWAS) data that uses genetic variation as an instrumental variable (IV) to proxy for exposure. The MR approach minimizes outcome bias due to common environmental confounders and reverse causality interference, and in recent years it has been used primarily to infer causality between exposure and outcome (13). The causal relationship between mtDNA copy number and Crohn’s disease has been previously studied by the MR method. However, the relationship between mtDNA copy number and other ADs has not been established by Mendelian randomization. There is also a lack of use of MR designs to explore reverse causality. Therefore, in the present study, we performed a Mendelian randomization study to assess the causal relationship between mtDNA copy number and ADs, including SLE, RA, AS, CD, UC, T1DM, thyrotoxicosis, psoriasis.

## 2 Methods

### 2.1 Two-sample MR

**Figure 1:**
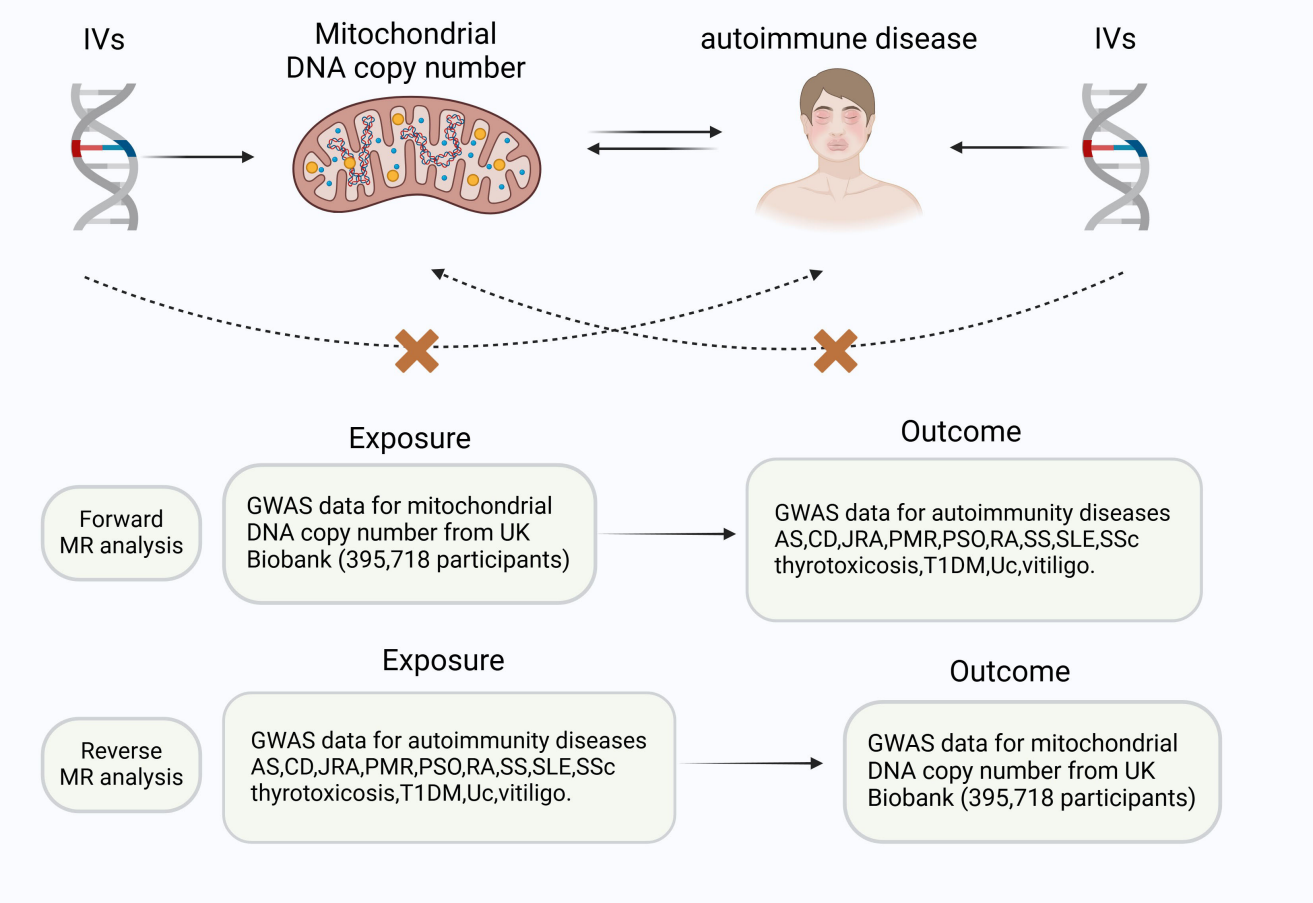
Three key principles of the bidirectional Mendelian randomization study design. IVs, instrumental variables.AS, ankylosing spondylitis,CD, Crohn’s disease, JRA, juvenile rheumatoid arthritis, PMR, polymyalgia rheumatica, PSO, psoriasis, RA, rheumatoid arthritis, SS, Sjogren’s syndrome, SLE, systemic lupus erythematosus, T1DM, type 1 Diabetes, UC, ulcerative colitis.

A two-sample MR study was conducted to assess whether there is a causal relationship between genetic susceptibility to mtDNA copy number and AD. Multiple single nucleotide polymorphisms (SNPs) were defined as instrumental variables (IVs). The three critical assumptions of the MR analysis were as follows (**Figure 1**): 1. IVs demonstrated a significant correlation with exposure; 2. IVs were not associated with any confounding factors, and 3. selected IVs had no direct link to the outcome variables (14).

### 2.2 Data sources

The summary statistics for mtDNA copy number from Chong et al.’s study (15)were obtained from the UK Biobank (16). The summary statistics patients with SLE (5,201 patients and 9066 controls) were obtained from the GWAS catalog (https://www.ebi.ac.uk/gwas/). Summary-level statistical data for other ADs were obtained from the FinnGen GWAS results. In the IEU Open GWAS program, the AD and GWAS IDs corresponding to AS, CD, juvenile rheumatoid arthritis (JRA), polymyalgia rheumatica (PMR), PSO, RA, Sjogren’s syndrome (SS), systemic sclerosis (SSc), thyrotoxicosis, T1DM, UC, and vitiligo were “finn-b-M13_ANKYLOSPON”, “finn-b-K11_CD_STRICT”, “finn-b-M13_JUVERHEU”,”finn-b-M13_POLYMYALGIA”, “finn-b-PSORI_STRICT”, “finn-b-M13_RHEUMA”, “finn-b-M13_SJOGREN”, “finn-b-M13_SYSTSLCE”,”finn-b-THYROTOXICOSIS”, “finn-b-T1D_WIDE”, “finn-b-K11_ULCER”, “finn-b-L12_VITILIGO”. The present study did not use GWASs for AD from the UK Biobank to avoid potential bias due to sample overlap.

### 2.3 Selection of IVs

For our analysis, SNPs significantly linked to the exposure (P < 5 × 10^−8) were selected as instruments, ensuring that they were linkage disequilibrium (LD) independent with an R^2^ of 0.001 and a 10,000 kb window size. In the reverse analysis, the criterion for P-value was relaxed (P < 5 × 10^−6) when using Finland GWAS to include more SNPs. SNPs associated with the outcome risk were excluded to avoid confounding effects. Furthermore, palindromic SNPs from the MR analysis were omitted to maintain the accuracy of the results.

### 2.4 Statistical analyses

We conducted the Mendelian randomization analysis using the “TwoSampleMR” packages in R software (version 4.2.2), applying methods such as inverse variance weighting (IVW) (17), MR-Egger (18), weighted median (19), and weighted mode (20) for causal inference. IVW was the primary method, with significance defined by a P-value < 0.05 and consistent results across all methods. P-values were adjusted using the false discovery rate (FDR) correction to control the false positive rate in multiple tests (21).

### 2.5 Sensitivity analyses

“Leave-one-out” sensitivity analyses to determine if a single SNP might skew causality results (22). Heterogeneity was evaluated using Cochran’s Q statistic (23), when heterogeneity was present, the random effects IVW test was applied for more conservative and reliable estimates.

### 2.6 Experimental verification

Adult patients meeting the 2010 classification criteria for RA of the American College of Rheumatology were recruited at the Second Affiliated Hospital of Nanchang University in 2024 with the approval of the Ethics Committee of the Second Affiliated Hospital of Nanchang University, and all patients participating in the study signed an informed written consent. RA activity was assessed by DAS28-ESR at blood collection, and CDAI scoring was performed by the attending physician.

Peripheral blood samples (4.5 mL) were collected in EDTA tubes via peripheral venipuncture. Within 4 hours of collection, genomic DNA, including mitochondrial DNA (mtDNA), was extracted from the whole blood using the Blood Genome DNA Extraction Kit (Takara, Cat. No. 9450). The DNA was precipitated with isopropanol, washed with 70% ethanol, and dissolved in TE buffer for further analysis. The relative mtDNA copy number was quantified using the Human Mitochondrial DNA Monitoring Primer Set (Takara, Cat. No. 7246) with TB Green^®^ Premix Ex Taq™ II (Tli RNaseH Plus) (Takara, Cat. No. RR820A) on Real-Time PCR System. The PCR reactions were prepared with 12.5 μL of TB Green Premix, 1 μL of each primer mix (10 μM), and 2 μL of DNA template in a final volume of 25 μL. The cycling conditions were as follows: initial denaturation at 95°C for 30 seconds, followed by 40 cycles of 95°C for 5 seconds and 60°C for 30 seconds. The relative mtDNA copy number was calculated using the ΔCt method. Specifically, ΔCt1 was calculated as the difference between the Ct values for SLCO2B1 (nuclear DNA) and ND1 (mtDNA), and ΔCt2 was calculated as the difference between the Ct values for SERPINA1 (nuclear DNA) and ND5 (mtDNA). The mtDNA copy number was determined by calculating 2^ΔCt for both ΔCt1 and ΔCt2, and the final mtDNA copy number was the average of these values. After obtaining the experimental results, we used Spearman rank order correlation coefficients to assess the relationship between mtDNA copy number and experimental subjects’ age, sex, disease duration month, ESR, CRP, DAS28ESR, DAS28CRP, SDAI and CDAI association strength and direction. In addition to this, two separate correlation analyses were conducted to explore the different association profiles under DAS28-ESR grouping and CDAI grouping, and the results of these analyses were visualized by correlation heatmaps and scatter matrix plots.

## 3 Results

### 3.1 Univariate MR analysis

**Figure 2:**
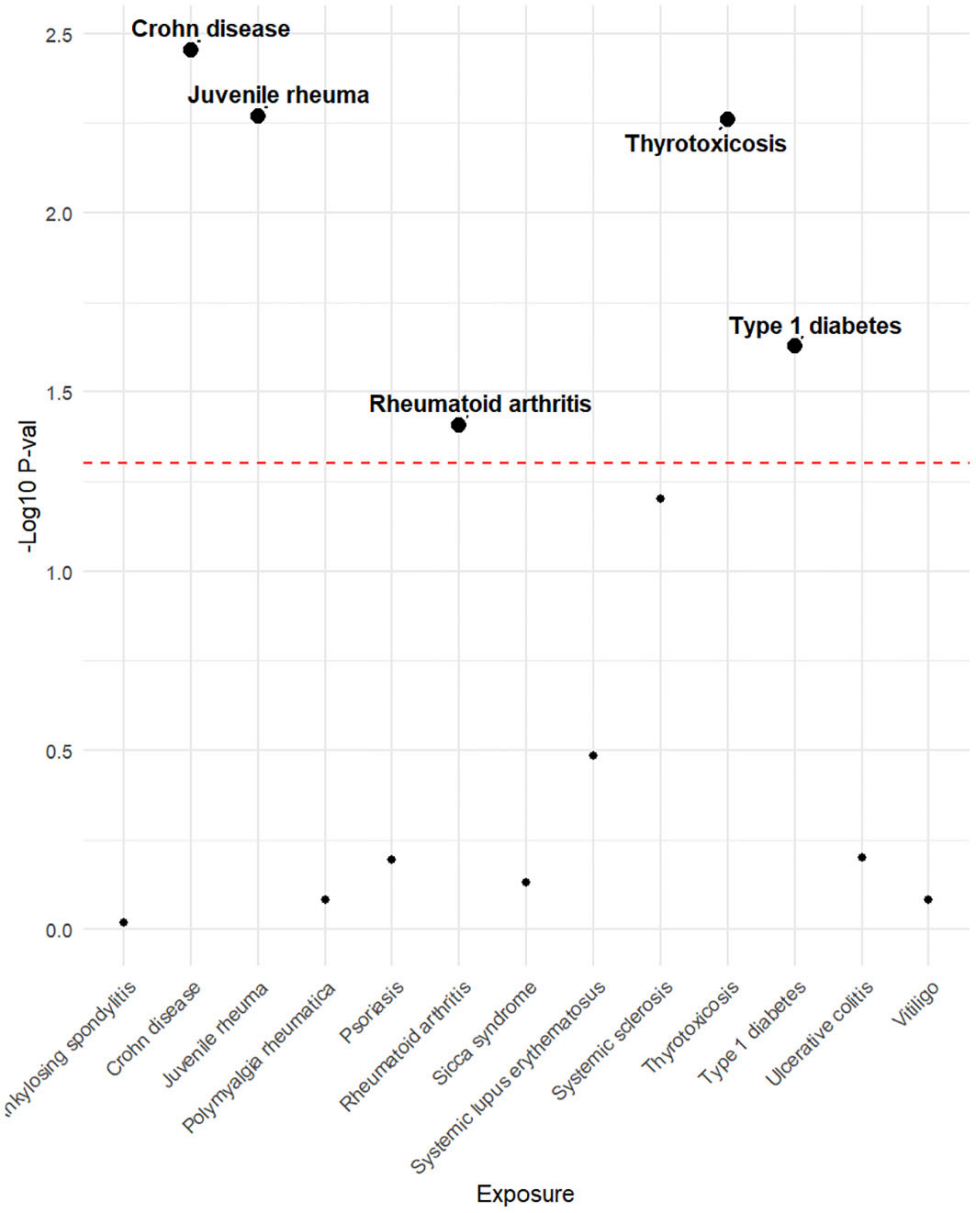
The P-value distribution of associations between mtDNA copy number and autoimmune disease in the Mendelian randomization analysis. The dashed line represents the threshold of the suggestive level of significance, set at P= 0.05.

**Figure 3:**
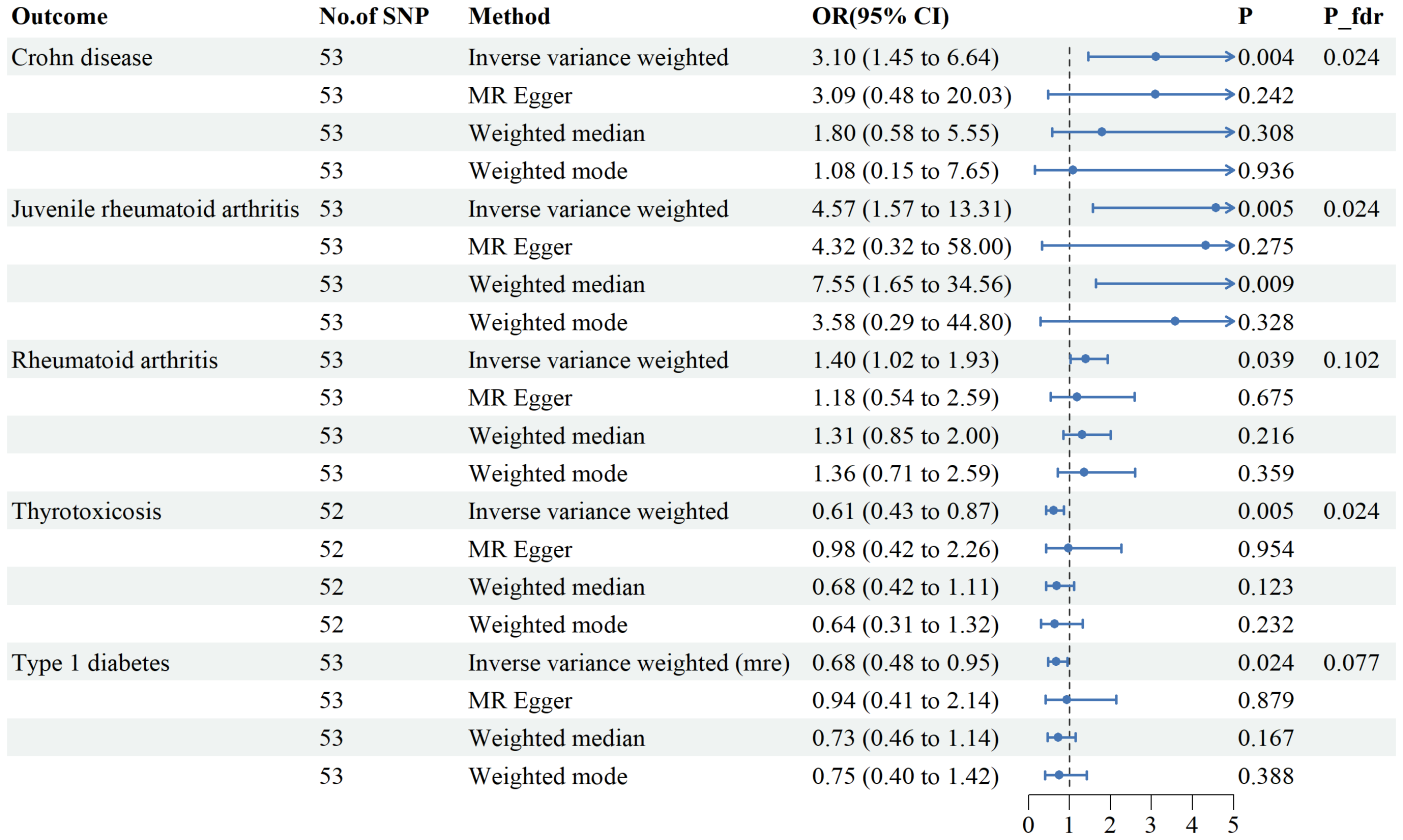
Associations between genetically predicted mtDNA copy numbers and five autoimmune diseases examined by four MR methods.MR, Mendelian randomization; IVW, inverse-variance weighted, CI, confidence interval.

**Figure 4:**
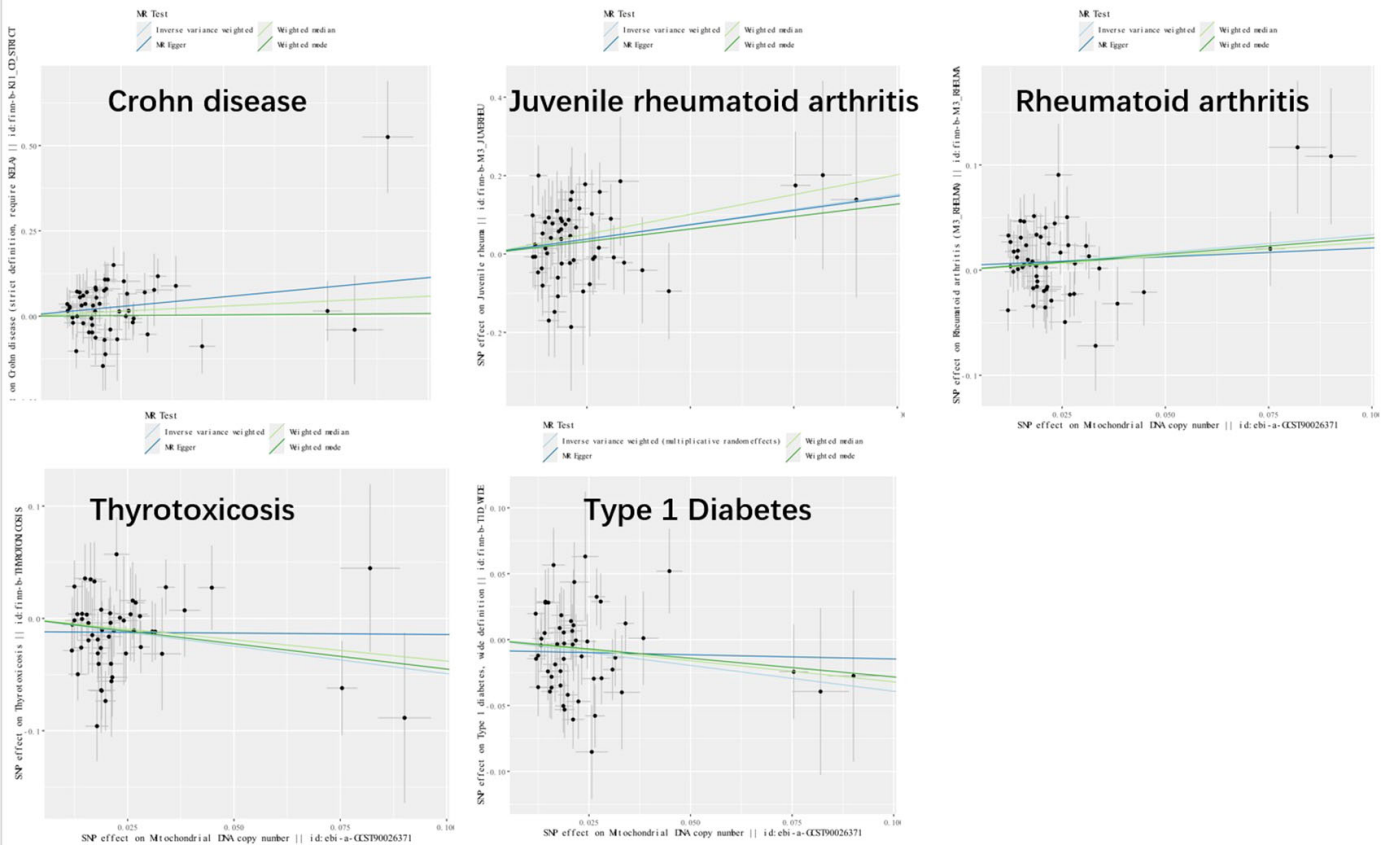
Scatter plot illustrating the causal effects of mtDNA copy number and five autoimmune diseases. SNP, single nucleotide polymorphism.

Genetic variants of mtDNA copy number were associated with CD (odds ratio [OR]=2.51, 95% confidence interval [CI] 1.56–4.22, P<0.001), JRA (OR=1.87, 95% CI1.17-7.65, P=0.022), RA (OR=1.71, 95%CI1.18-2.47, P=0.004), thyrotoxicosis (OR=0.51, 95% CI0.27-0.96, P=0.038), and T1DM (OR=0.51, 95% CI0.27-0.96, P=0.038) (**Figures 2**, **3**). After applying FDR correction, the adjusted P values for the IVW model of CD, JRA, and thyrotoxicosis remained below 0.05, further strengthening the reliability of our conclusions. **Figure 4** presents a scatter plot delineating the inferred causal linkages between mtDNA copy number and ADs. The potential heterogeneity (**Supplementary Table 1**) was also analyzed. Based on MR-Egger’s examination of the intercept term, the causal analysis did not show significant evidence of horizontal pleiotropy (**Supplementary Table 1**). The IVs used are shown in **Supplementary Table 3**.

### 3.2 Reverse MR analysis

**Figure 5:**
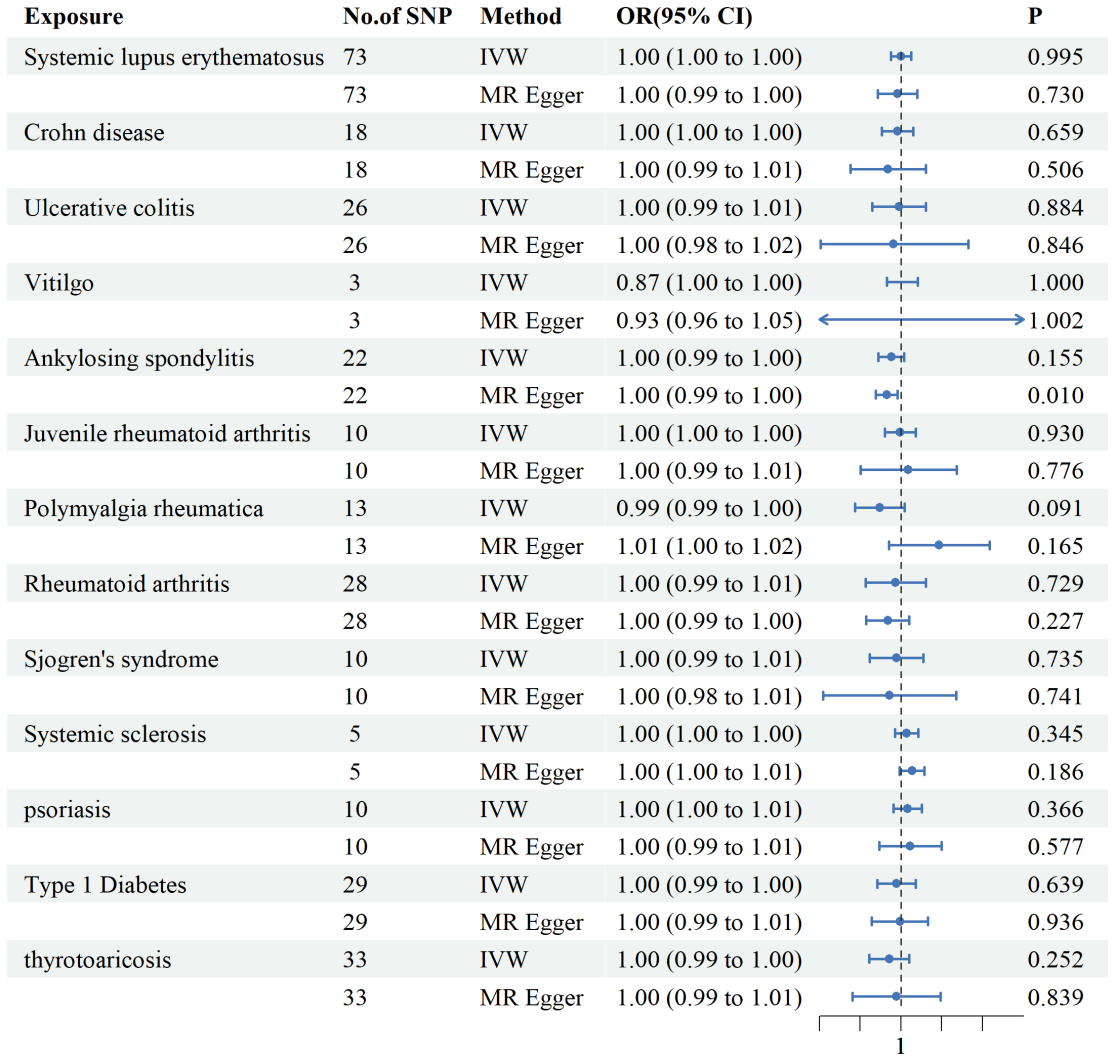
Associations between genetically predicted autoimmune diseases and mtDNA copy number examined using four MR methods.MR, Mendelian randomization; IVW, inverse-variance weighted, CI, confidence interval.

ADs were examined as exposures, and mtDNA copy numbers as the outcomes (**Figure 5**). we included Seventy-three SNPs for SLE, 18 for CD, 26 for UC, 3 for Vitiligo, 22 for AS, 10 for JRA, 13 for PMR, 28 for RA, 10 for SS, 5 for SSc, 10 s for PSO,29 for T1DM, and 33 for thyrotoxicosis. According to the IVW method, no substantial associations were found. Horizontal pleiotropy was not observed in any of the sensitivity analyses (**Supplementary Table 2**). The IVs used are shown in **Supplementary Table 4**.

### 3.3 Experimental verification

**Figure 6:**
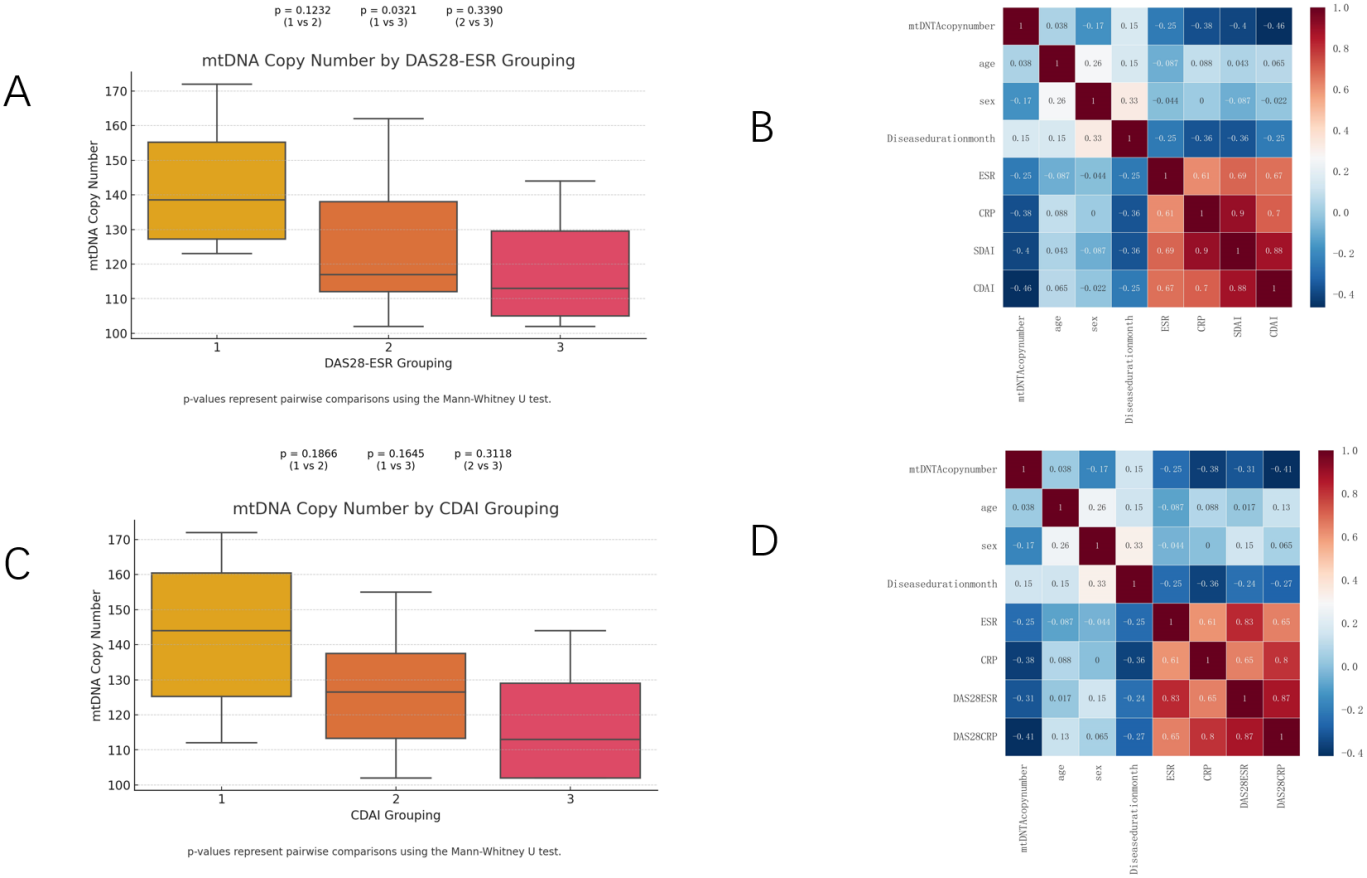
mtDNA copy numbers **(A, C)** in RA patients throughout all strata of DAS28-ESR and CDAI activity. Heatmap **(B, D)** showing the strength and direction of correlations between mtDNA and age, sex, disease duration month, ESR, CRP, DAS28ESR, DAS28CRP, SDAI and CDAI for different subgroups in Spearman correlation analysis.

Twenty-four patients with RA were recruited and the mean DAS28-ESR was 3.98 and the mean CDAI was 13.41 (**Supplementary Table 5**). The mtDNA copy number was compared among patients stratified by DAS28-ESR grouping (Group 1, Group 2, and Group 3). The mtDNA copy number was calculated for patients stratified by DAS28-ESR groupings. **Figure 6** shows the results. The mean mtDNA copy number in Group 1 (DAS28-ESR ≤ 3.2) was 142, representing the highest mean value among the groups. In Group 2 (3.2 < DAS28-ESR ≤ 5.1), the mean mtDNA copy number was126.44. Similarly, in Group 3 (DAS28-ESR > 5.1), the mean mtDNA copy number was 118.29. The results indicate no significant differences between Group 1 and Group 2, as well as between Group 2 and Group 3.A statistically significant difference was observed between Group 1 and Group 3 (p = 0.0321), suggesting a lower mtDNA copy number in patients in Group 3 compared to Group 1. Similar comparisons were made across CDAI groupings (Group 1, Group 2, and Group 3). The analysis revealed no statistically significant differences between Group 1, Group 2 and Group 3. Combining the results of **Figures 6B, D** and **Supplementary Table 3**, we can further see the direction and strength of correlation between mtDNA copy number and other variables. As shown, mtDNA copy number was positively correlated with RA replacement disease duration, whereas the correlations with gender and inflammation-related indicators such as ESR, CRP, DAS28-ESR, DAS28-CRP, SDAI, and CDAI were negative. The heatmap module representing the relationship between DAS28-CRP, CDAI and mtDNA copy number was the darkest in color, implying a statistically significant negative correlation of mtDNA copy number only with DAS28-CRP (p=0.044) and CDAI (p=0.023).

## 4 Discussion

To the best of our knowledge, this is the first study using MR to fully explore the relationship between mtDNA copy number and ADs. Our study revealed potential associations between genetically predicted mtDNA copy number and CD, JRA, RA, thyrotoxicosis and T1DM. In addition, we validated this in patients with RA and found that whole blood mtDNA copy number decreased with increasing disease activity.

Previous observational studies have explored the relationships between mtDNA copy number and various ADs including AS (10), CD (11, 24), JRA (25), PSO (12),RA (9), SS (26), SLE (6, 27–29), SSc (7, 30), thyrotoxicosis (31), T1DM (8), UC (11, 32, 33), and vitiligo (34). Studies on SLE have shown that mtDNA copy number correlates with disease activity, response to glucocorticoids, and prognosis (6, 27). Regarding SSc, observational studies have yielded conflicting results, with some showing negative correlations (30)and others showing nonsignificant correlations (7). The same is valid for PSO. Previous clinical articles have found lower mtDNA levels in patients with PSO than controls (12), other research has indicated increased mtDNA copy number in patients with PSO. Basic research has shown that mitochondrial dysfunction contributes significantly to the pathogenesis of PSO by modulating innate immunity via redox-sensitive inflammatory pathways (35, 36). A study from the UK on 60 patients with UC demonstrated that elevated mtDNA levels correlating with C-reactive protein, endoscopic severity, and disease activity. However, our results did not show a bidirectional causal relationship between mtDNA copy number and these diseases. Oxidative stress is known a key factor in the pathogenesis of CD and as a key mechanism leading to tissue and cellular damage (37, 38). A previous meta-analysis of MR studies revealed a robust causal relationship between mtDNA copy number and CD (39), which is consistent with our results. Most current clinical studies on mtDNA copy number and ADs are cross-sectional or retrospective, which making them prone to reverse causality and confounding factors. Our MR study provides ideas for the design of future clinical studies. Interventions targeting mitochondrial function may be a new strategy for treating these ADs. For example, modulating mitochondrial function could help reduce inflammatory responses and alleviate disease symptoms (40). Such investigations might pave the way for innovative clinical tests to enhancing the diagnosis and prognosis of ADs or improve patient stratification to enable more tailored clinical care approaches (41).

In recent years, there has been growing interest in the role of mitochondria in ADs, especially the influence of mitochondrial DNA on the onset and progression of such diseases. Many studies have demonstrated that alterations in mtDNA copy number contribute to developing of ADs. One way mtDNA affects autoimmunity is by alter mitochondrial function, thereby increasing oxidative stress and damaging tissues and cells. Previous basic research on RA has shown that oxidative stress plays an important role in the pathogenesis of RA (42–44). Interestingly, our study revealed stronger relationship between JRA and mtDNA copy number than with RA, providing a new direction for exploring the mechanism of JRA. Moreover, mtDNA mutations can alter cellular energy metabolism, thereby altering intracellular ATP levels and affecting the onset and progression of ADs (45, 46). The purinergic system is involved in a variety of pathological and physiological processes and plays a role in the pathophysiology of RA (47). Under physiological conditions, extracellular ATP is present at approximately 10 nM, which is regulated by nucleic acid exonucleases anchored to the plasma membrane (48). However, during pathological events, the concentration of extracellular ATP can rise significantly, reaching micromolar levels (49).. When stress induces cellular damage, ATP is released, acting as a damage-associated molecular pattern (DAMP) to initiate a pro-inflammatory response. DAMPs are an endogenous factors that specifically recruit and activate innate immune cells to promote healing and tissue homeostasis (50).In autoimmune disorders such as RA, failure to regulate DAMPs exacerbates inflammation (51). DAMPs act as ATPs that play crucial roles, through positive feedback, facilitating the early inflammatory process in RA and prolonging immune activation. Mitochondria can respond to external or endogenous stresses by triggering mitochondrial autophagy, mtDNA release, and apoptosis, and can ultimately regulating the survival or death of stressed cells (52–54). The release of mtDNA into the cytoplasm results in the expression of type I interferon (IFN) and a variety of inflammatory cytokines, including members of the TNF superfamily, interleukins and chemokines (55). Excessive release of mtDNA under sterile conditions triggers an IFN response leading to autoinflammatory conditions such as SLE (56, 57). Moreover, a study on mitochondrial transplantation showed that the *in vitro* transfer of healthy mitochondria into CD4+ T cells from patients with RA restored aspartate metabolism and reduced TNF production (58, 59). It has also been noted that in patients with JRA, mitochondrial-encoded DNA expression is inhibited, and the specific downregulation of mitochondrial-encoded components suggests a possible disruption of mitochondrial respiratory function, which is critical for cellular energy production and immune response (25). These studies imply that mitochondria are a potential therapeutic target that could provide more precise and effective patient treatment.

This study is the first comprehensive exploration of the reverse causality between ADs and mtDNA copy number. The onset and progression of ADs involve oxidative stress and inflammatory responses, making it mechanistically plausible that ADs may further affect mtDNA copy number. However, in the present study, we did not find any causal relationship between ADs and mtDNA copy number. This may be related to the fact that the effects of ADs on mtDNA copy number involve multiple complex pathways that are difficult to capture adequately in current studies. In reverse Mendelian Randomization studies, the relatively small number of cases in comparison to a larger pool of controls might have obstructed the identification of genetic variations linked to the disease. Such an imbalance could potentially bias the results of the study and obstruct an accurate determination of the relationship between genetic variations and the disease (60).

Our study has several advantages. First, a two-sample MR method was employed using SNPs as instrumental variables, which were less susceptible to confounding factors and reverse causality due to the random assignment during meiosis. Second, this is the first MR study to systematically assess the causal relationship between mtDNA copy number and ADs, and it is the first to report the causal relationship between mtDNA copy number and certain ADs from an MR perspective. A better understanding of the relationship between mtDNA and AD could contribute to a clearer understanding of the pathophysiology of ADs and help in the search for new biomarkers. Nevertheless, our study has several limitations. Firstly, it focused only on GWAS data from a European population, limiting its generalizability to the population. Genetic variations and environmental interactions can vary significantly across populations, which may affect the applicability of our results to non-European populations. Further studies in diverse populations are needed to confirm our findings. This will help in understanding population-specific genetic architectures and improve the precision of personalized medicine approaches. Second, due to the limitations of MR study, the second and third major assumptions are difficult to ensure precisely. Therefore, we performed MR-Egger to detect pleiotropy, which did not show pleiotropy. Third, our GWAS was derived from two samples, and data from different sources may have different measurements or definitions, which may have affected the accuracy of the results.

In conclusion, our study suggests that mtDNA copy number may affect several ADs, such as CD, JRA and T1DM. Further clinical and basic studies are needed to understand the relationship between mtDNA and ADs in the future.

## Data Availability

The original contributions presented in the study are included in the article/**Supplementary Material**. Further inquiries can be directed to the corresponding author.
