# Supplementary material for: The role of mitochondrial DNA copy number in autoimmune disease: a bidirectional two sample mendelian randomization study
Source: Front Immunol. 2024 Oct 9;15:1409969. doi: 10.3389/fimmu.2024.1409969 (PMC11502960; doi:10.3389/fimmu.2024.1409969)
Supplement: Supplementary file 1 [file DataSheet1.docx]

**Supplementary Figure 1. Forest plot of causal effects of mtDNA-CN on five significant autoimmunity disease in forward MR analysis**


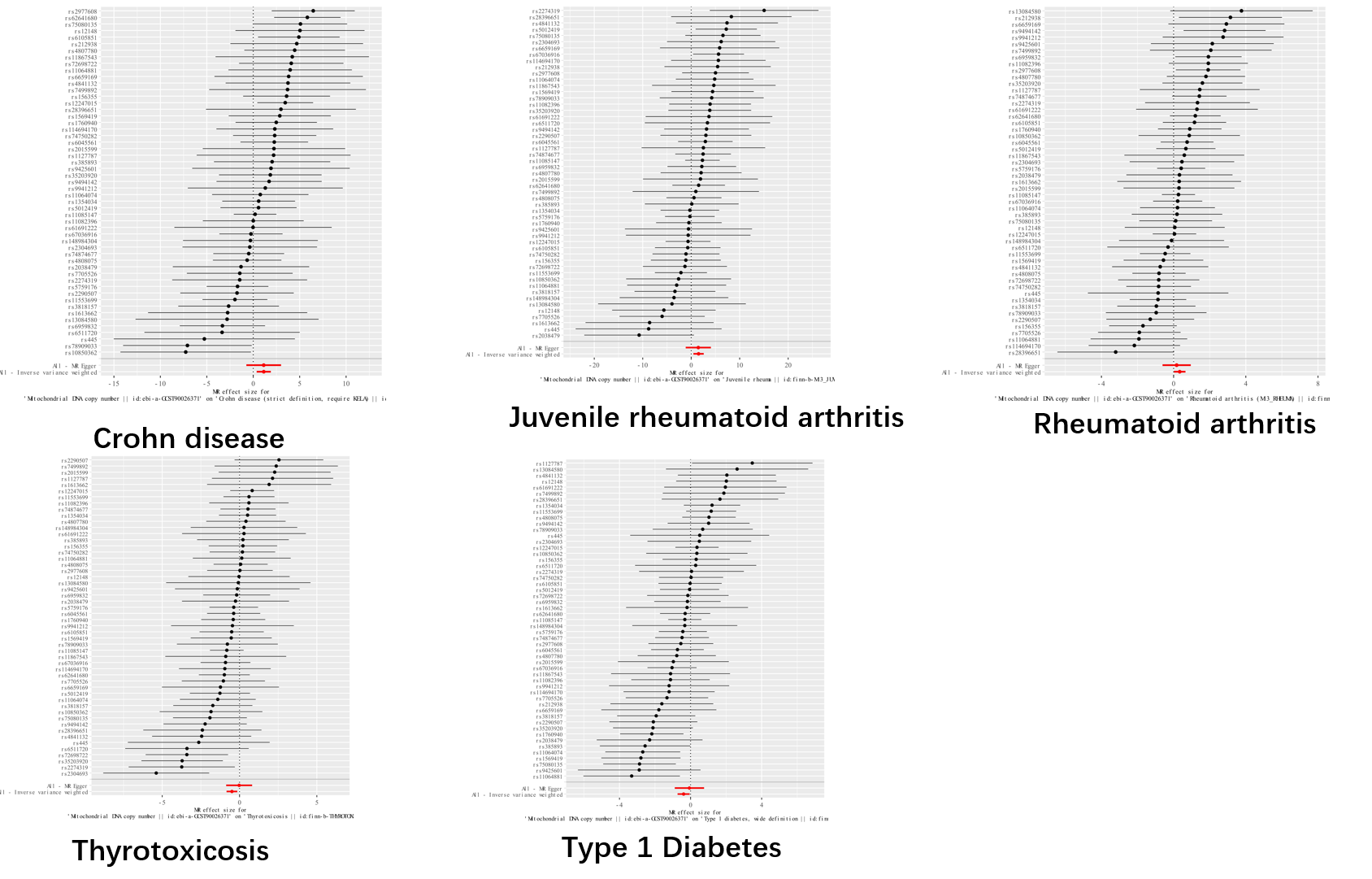


**Supplementary Figure 2. Leave-one-out plots of the causal effect of mtDNA-CN on five autoimmunity disease in forward MR analysis**


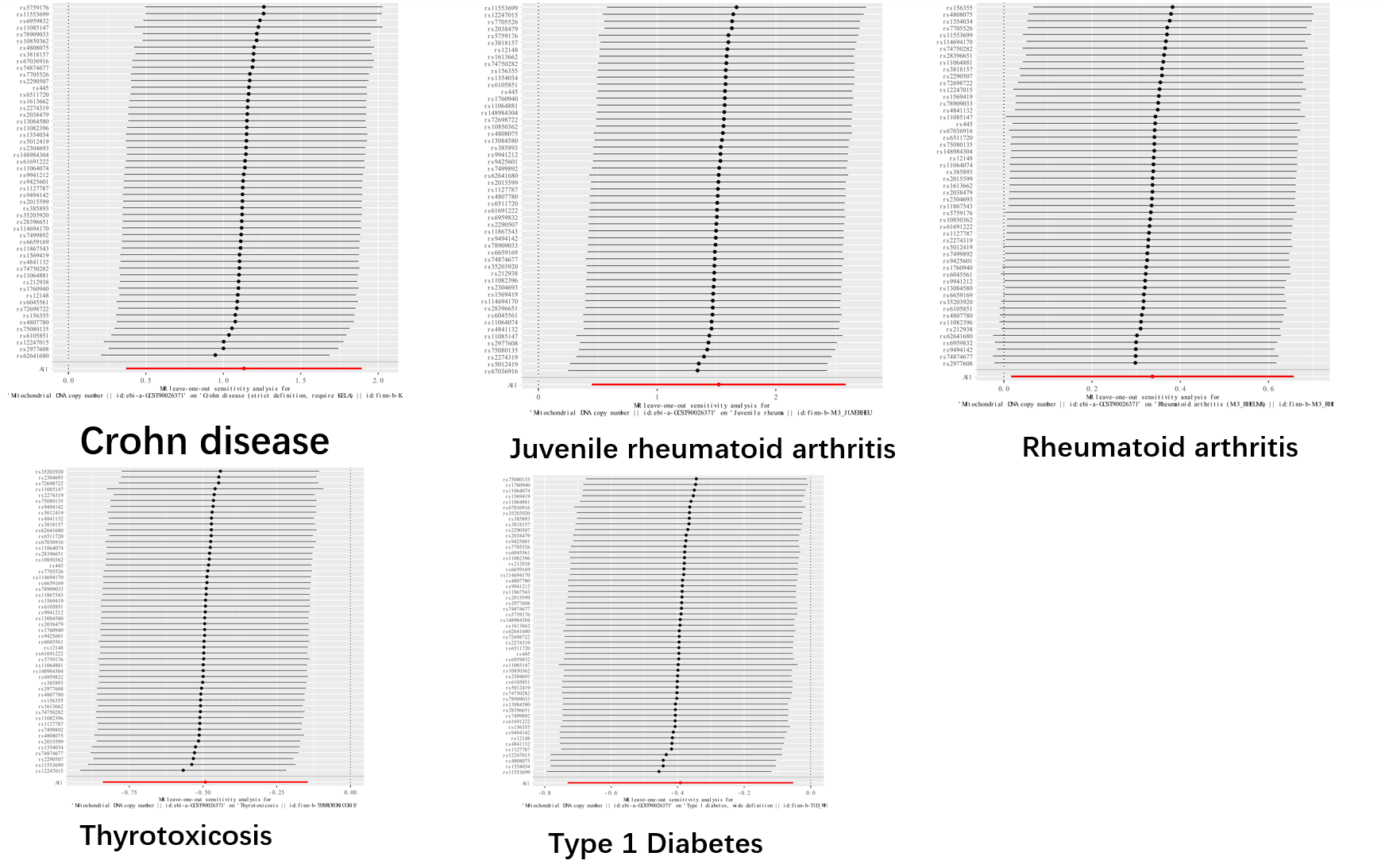


**Supplementary Figure 3. Funnel plot of the causal effect of mtDNA-CN on five autoimmunity disease in forward MR analysis**


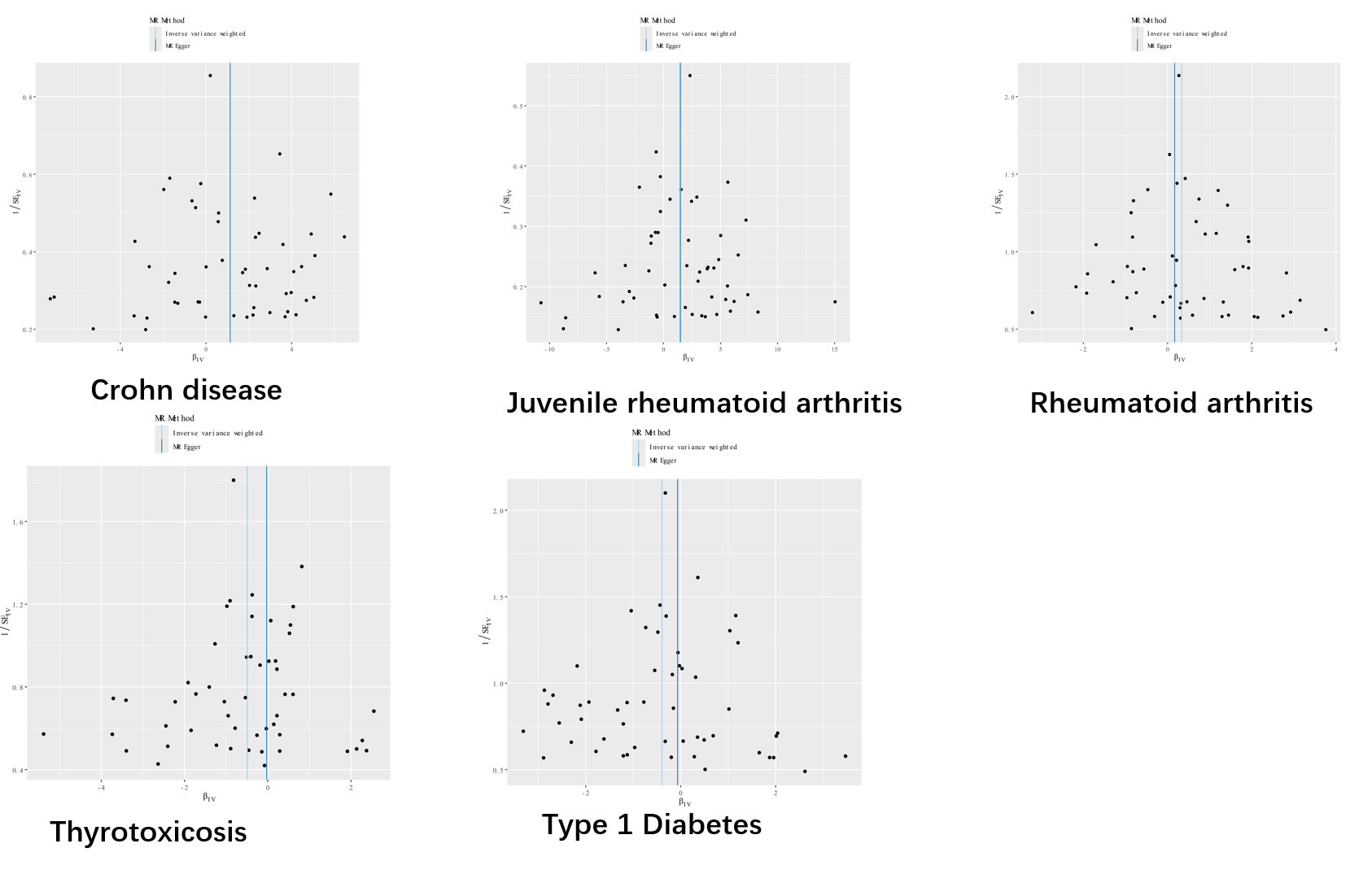


**Supplementary Figure 1. Leave-one-out plots of causal effects of autoimmunity disease on mtDNA-CN in reverse MR analysis**

**
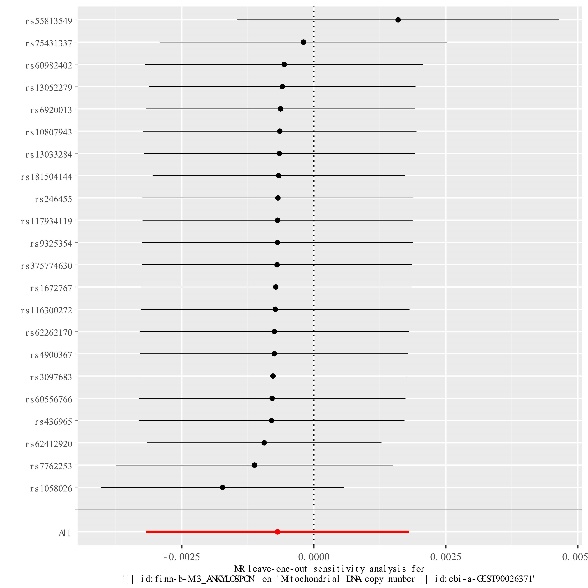
 Ankylosing Spondylitis on mtDNA-CN**

**
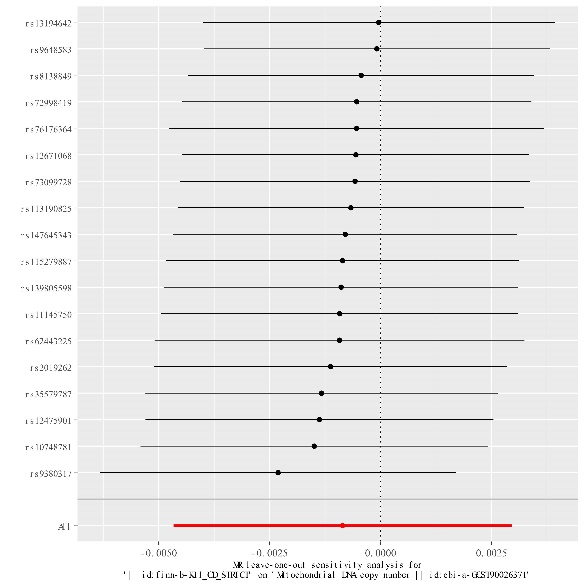
** **Crohn's disease on mtDNA-CN**

**
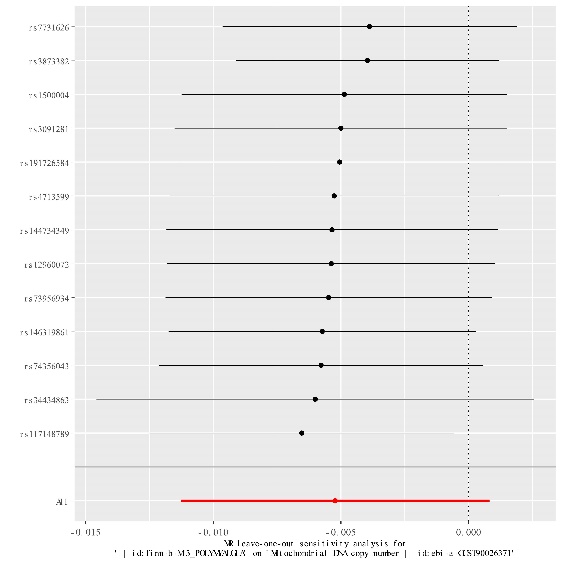
** **polymyalgia rheumatica on mtDNA-CN**

**
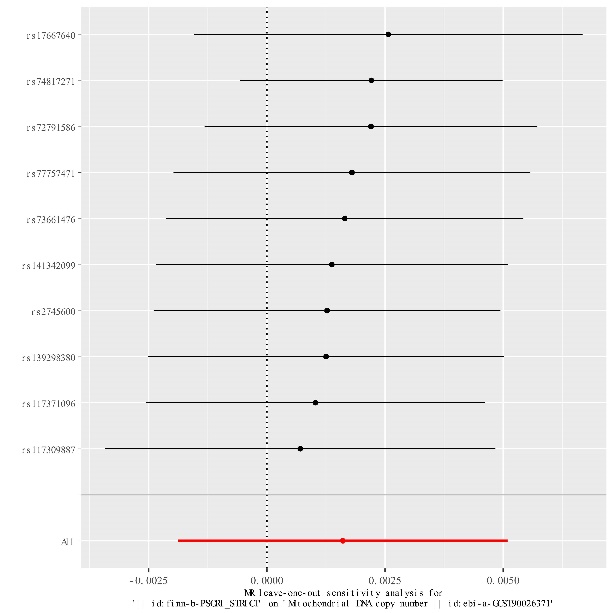
**  **psoriasis on mtDNA-CN**

**
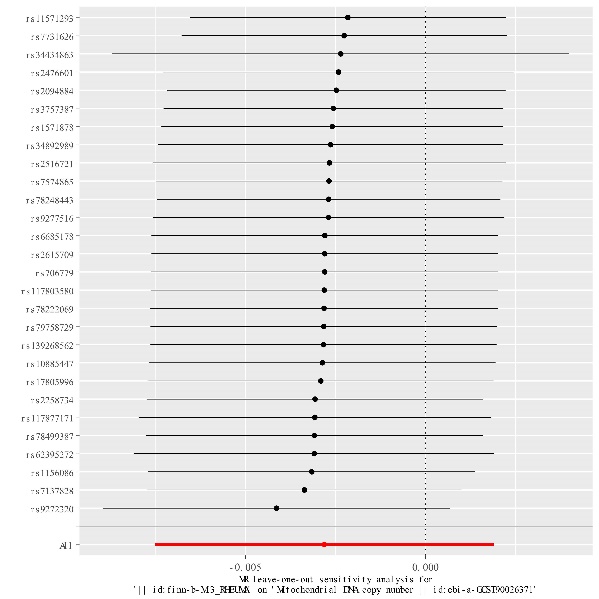
** **rheumatoid arthritis on mtDNA-CN**

**
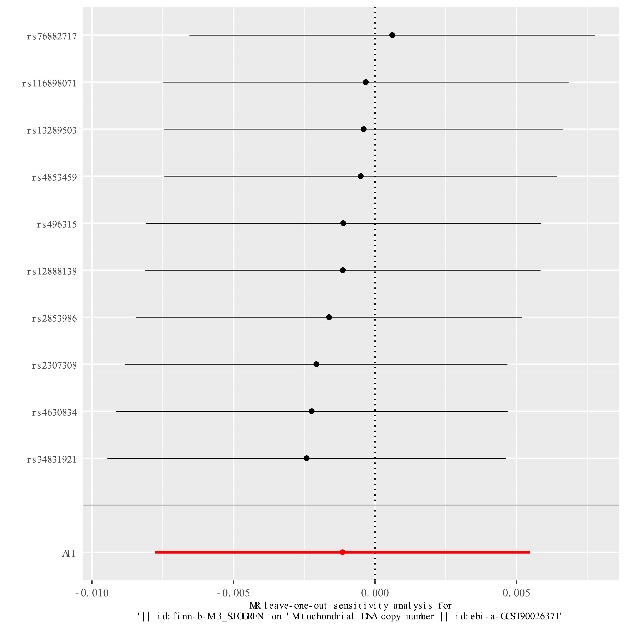
** **Sjogren's syndrome on mtDNA-CN**

**
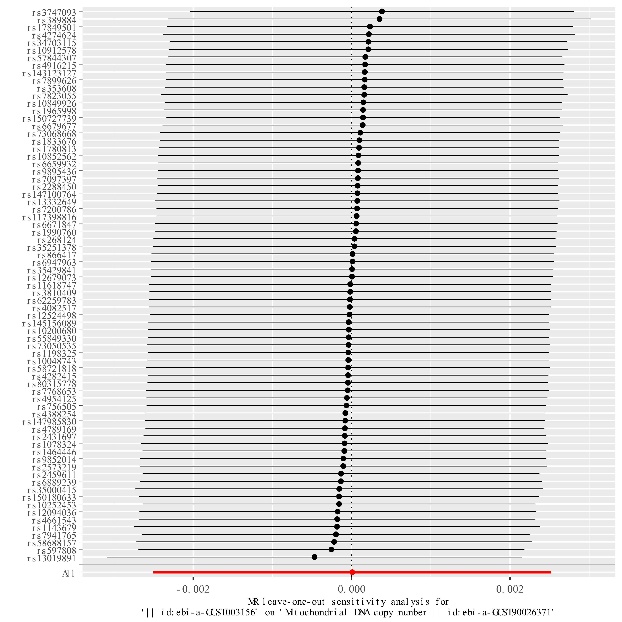
** **SLE on mtDNA-CN**

**
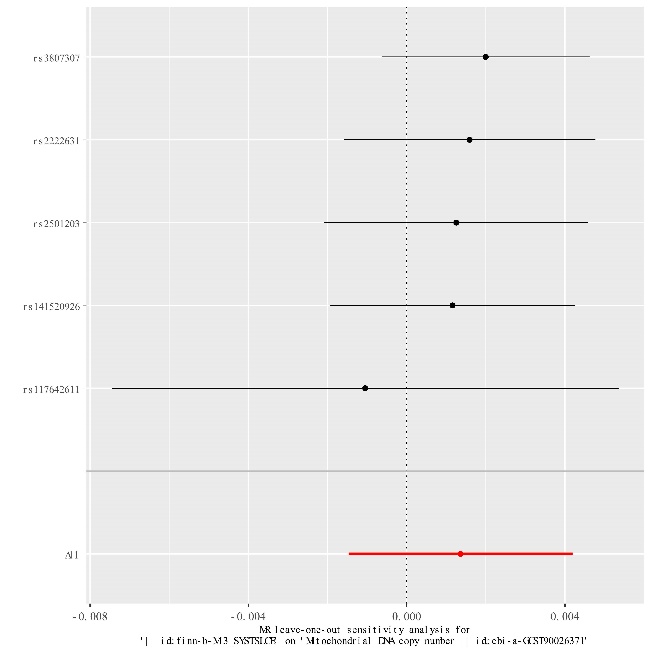
**

**systemic sclerosis on mtDNA-CN**

**
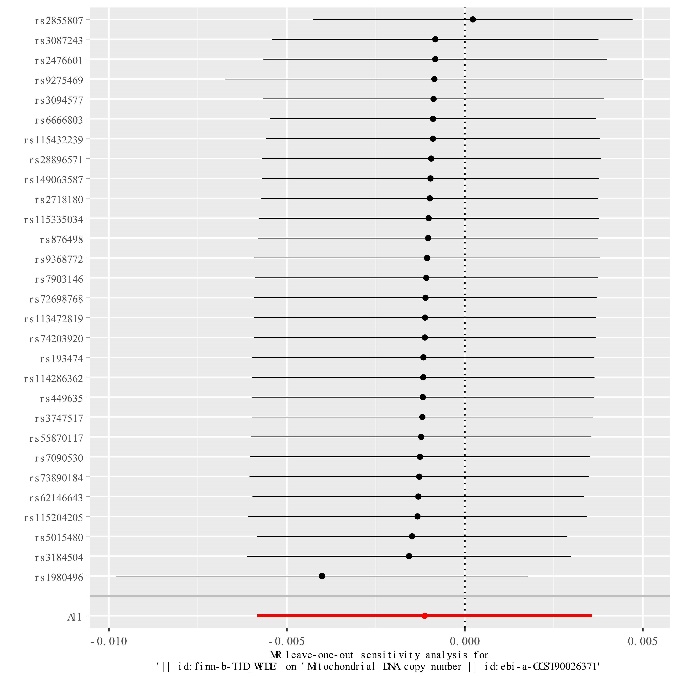
** **Type 1 diabetes on mtDNA-CN**

**
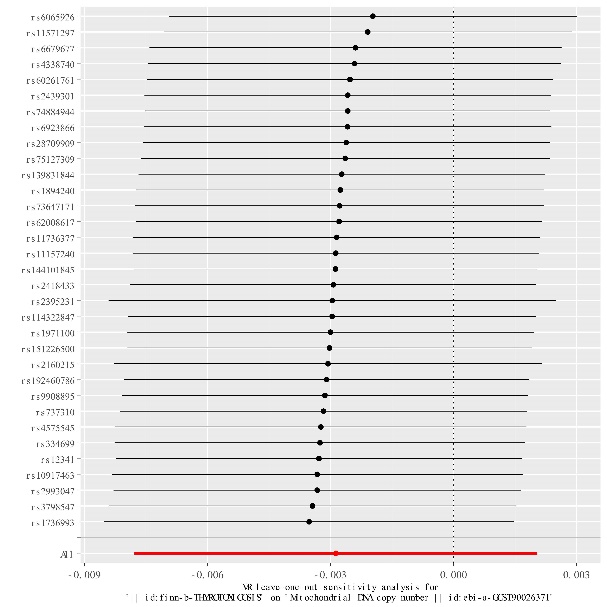
 thyrotoaricosis on mtDNA-CN**

**
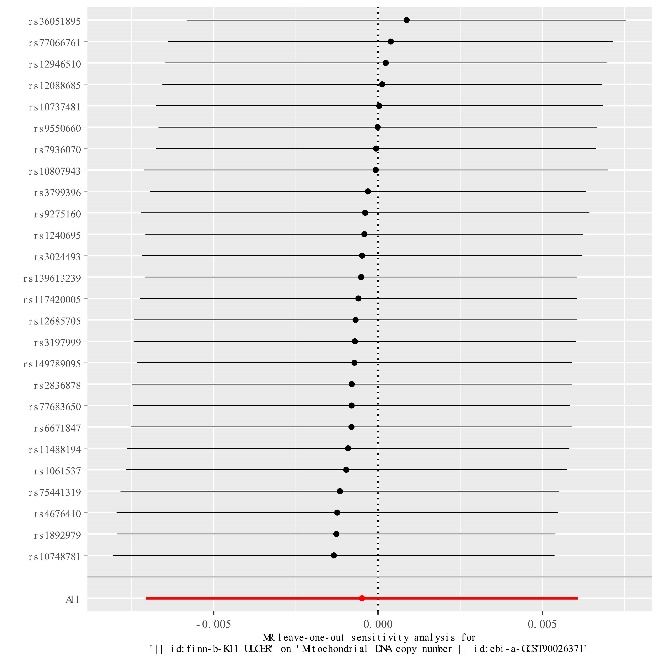
** **ulcerative colitis on mtDNA-CN**

**
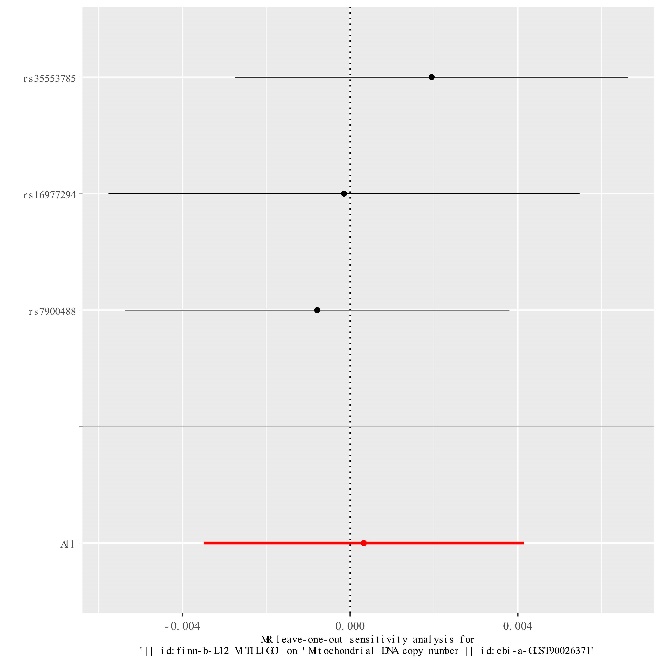
Vutiligo on mtDNA-CN**
